# Abduction and adduction malpositioned lateral radiographs cannot be used for proper assessment of the posterior tibial slope

**DOI:** 10.1002/ksa.70127

**Published:** 2025-10-28

**Authors:** Moses Kamal Dieter El Kayali, Luis Vincent Bürck, Stephen Fahy, Alan Getgood, Clemens Gwinner, Lorenz Pichler

**Affiliations:** ^1^ Center for Musculoskeletal Surgery Charité—Universitätsmedizin Berlin Berlin Germany; ^2^ Department of Orthopaedic Surgery Sporthopaedicum Berlin Germany; ^3^ Department of Orthopaedic Surgery ASPETAR, Orthopaedic and Sports Medicine Hospital Doha Qatar; ^4^ Department of Orthopedics and Trauma‐Surgery Medical University of Vienna Vienna Austria

**Keywords:** anterior cruciate ligament, lateral knee radiograph, medial posterior tibial slope, PTS

## Abstract

**Purpose:**

To compare medial posterior tibial slope (mPTS) measurements on abduction/adduction malpositioned versus properly positioned lateral knee radiographs within the same patients. We hypothesized that malpositioning would significantly alter mPTS values and potentially change surgical classification at the commonly used 12° threshold for slope‐correcting osteotomies.

**Methods:**

This retrospective analysis included patients with preoperative knee radiographs between October 2020 and March 2023 at a single academic institution. Patients were included if at least two lateral knee radiographs were available: one with abduction/adduction malalignment (proximal‐distal distance [PDD] ≥5 mm) and one properly aligned (PDD < 5 mm). Radiographs with insufficient tibial length, rotational malalignment, or prior osseous surgery or fractures were excluded. mPTS was measured using the method of Dejour & Bonnin. Inter‐ and intra‐rater reliability were assessed via intra‐class correlation coefficients (ICC > 0.9 for all comparisons). A *t*‐test was used to compare mPTS values. Cases with a measurement difference of ≥2° were identified. Bland–Altman analysis was performed, and classification shifts across the 12° mPTS threshold for slope‐correcting osteotomies were reported.

**Results:**

Eighty‐eight lateral knee radiographs from 44 patients (57% female) were analyzed. The mean mPTS was 7 ± 3° in properly aligned radiographs and 9 ± 3° in malaligned radiographs (*p* < 0.001), with a mean difference of 2°. In 59.1% of patients, mPTS differed by ≥2° between imaging conditions. Bland–Altman analysis showed 95% limits of agreement from –3.62° to 7.80°. In 15.9% of patients, classification relative to the 12° mPTS threshold changed.

**Conclusions:**

Abduction/adduction malalignment significantly alters mPTS measurements on lateral knee radiographs. A clinically relevant difference of ≥2° was observed in over half of patients, and 15.9% changed classification relative to the 12° surgical threshold. Lateral radiographs with a PDD ≥ 5 mm should not be used for surgical planning, and repeat imaging should be considered when mPTS values approach surgical decision thresholds.

**Level of Evidence:**

Level III diagnostic study.

**Abbreviations**
ACL: anterior cruciate ligament
APD: anterior–posterior distance
BMI: body mass index
ICC: intra‐class correlation coefficient
IQR: interquartile range
MAE: mean absolute error
MCID: minimal clinically important difference
mPTS: medial posterior tibial slope
PDD: proximal‐distal distance
PTS: posterior tibial slope
RMSE: root mean squared error
SD: standard deviation
SDC: smallest detectable change
SEM: standard error of measurement

## INTRODUCTION

The posterior tibial slope (PTS) is a surgically modifiable anatomical parameter that has been identified as a risk factor for anterior cruciate ligament (ACL) injury and graft failure in adolescents and adults [4, 12, 18, 29, 35]. Although high‐level evidence for a universal cut‐off is lacking, a PTS greater than 12° [1, 6, 20] with target PTS values between 4° and 7° is commonly used in clinical practice [4, 13, 34]. Accurate measurement of the PTS is therefore critical given its relevance for surgical decision‐making.

A recent international expert Delphi consensus statement highlighted several technical factors that may influence measurement accuracy [34]. While tibial length (≥15 cm) and rotational malalignment have long been recognized as major sources of error, attention to abduction and adduction malalignment has only recently increased [2, 11, 14, 22, 33]. Experimental work by Bixby [2] and, more recently, a biomechanical study by Peez et al. [26] demonstrated that both rotational (internal/external) and coronal (varus/valgus) malpositioning significantly affect the accuracy of PTS measurements. However, clinical data confirming these findings are currently lacking.

Therefore, the purpose of this study was to compare mPTS measurements on abduction/adduction malaligned and properly aligned lateral radiographs within the same patients. We hypothesized that mPTS values would differ significantly between the two imaging conditions and that such differences could alter surgical decision‐making when applying the 12° threshold for slope‐correcting osteotomies. This is clinically relevant, as misclassification based on malaligned radiographs may lead to inappropriate surgical indication.

## MATERIALS AND METHODS

This study was approved by the local institutional ethics committee (Nr. EA2/016/21) and conducted in accordance with the principles of the Declaration of Helsinki. Written informed consent was obtained from all participants prior to inclusion. All subjects were informed about the study objectives, procedures and potential risks.

### Patients

In this retrospective analysis, a total of 481 patients who underwent knee surgery at our academic orthopaedic surgery center between October 2020 and March 2023 were initially screened. Eligible patients had at least two lateral knee radiographs acquired during routine preoperative imaging: one demonstrating abduction/adduction malalignment, defined as a proximal‐distal distance (PDD) of ≥5 mm, and one with minimal or no abduction/adduction malalignment, defined as a PDD of <5 mm. Inclusion of both properly aligned and malaligned radiographs was possible because repeat imaging was frequently performed in clinical routine, either as quality‐control repeats during the same imaging session or as part of scheduled follow‐up examinations. If more than two eligible radiographs were available for the same patient, the pair with the shortest interval between the properly aligned and malaligned radiograph was selected to minimize potential temporal bias. Radiographs were categorized into two groups according to the 5 mm PDD cut‐off, which was proposed by Bixby et al. [2] as a clinically relevant threshold for PTS measurement accuracy. Additional inclusion criteria were availability of written patient consent and complete patient records.

Patients were excluded based on the following criteria: inadequate tibial shaft visualization on lateral radiographs (<15 cm of tibial shaft length [34]); rotational malalignment, defined as an anterior‐posterior distance (APD) of ≥5 mm [10, 11, 16, 24] (Figure 1); more than 6 months between the two radiographs; history of prior bony surgery or fractures involving the knee; incomplete or missing patient records; or lack of written informed consent. For all included patients, clinical records were reviewed to confirm that no interventions or clinical events occurred between imaging sessions that could have influenced posture or arthrokinematics. The patient inclusion process is shown in Figure 2.

**Figure 1:**
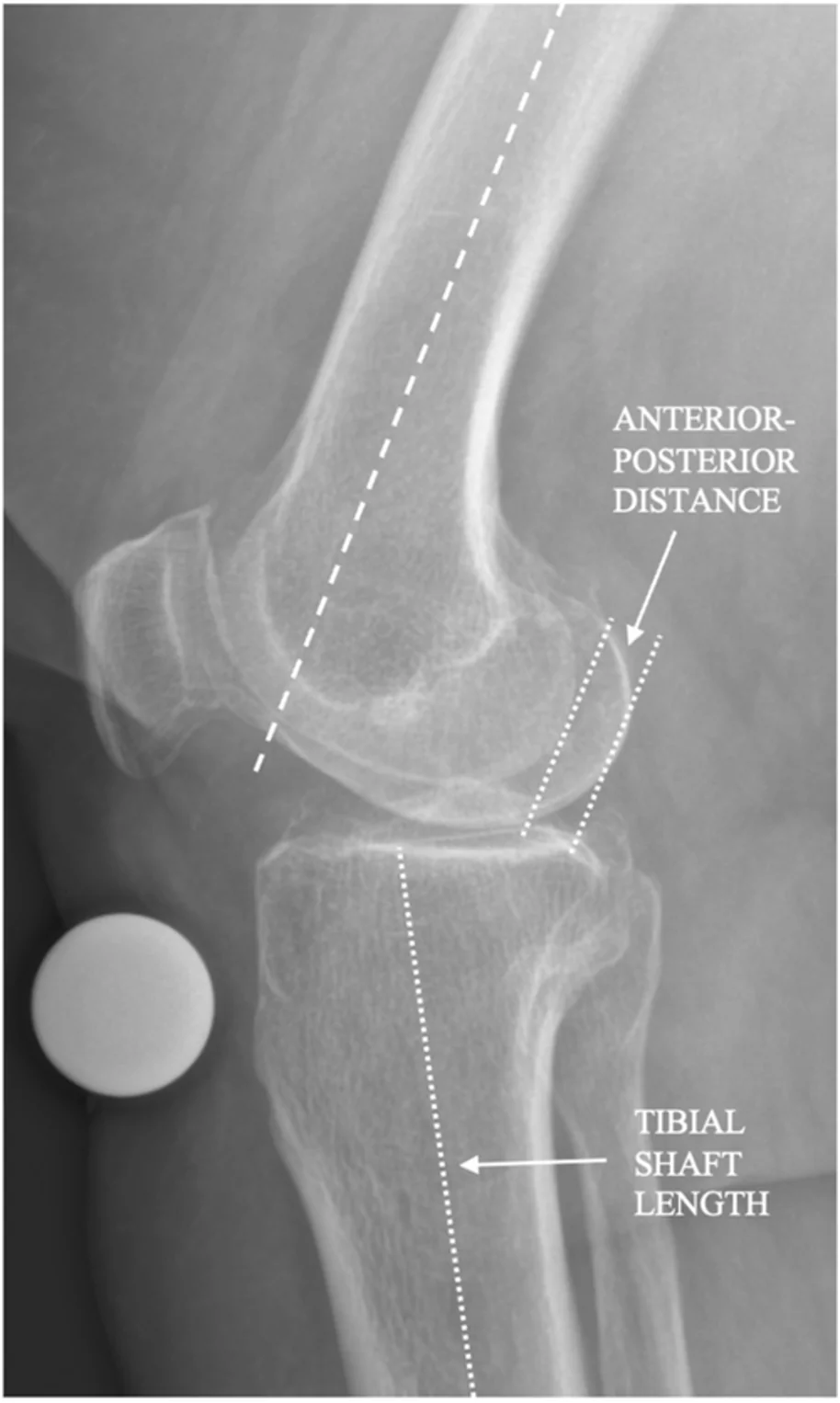
Radiographic exclusion criteria. Lateral radiograph of a right knee demonstrating exclusion due to rotational malalignment (APD ≥ 5 mm) and insufficient tibial shaft visualization (<15 cm). APD, anterior–posterior distance.

**Figure 2:**
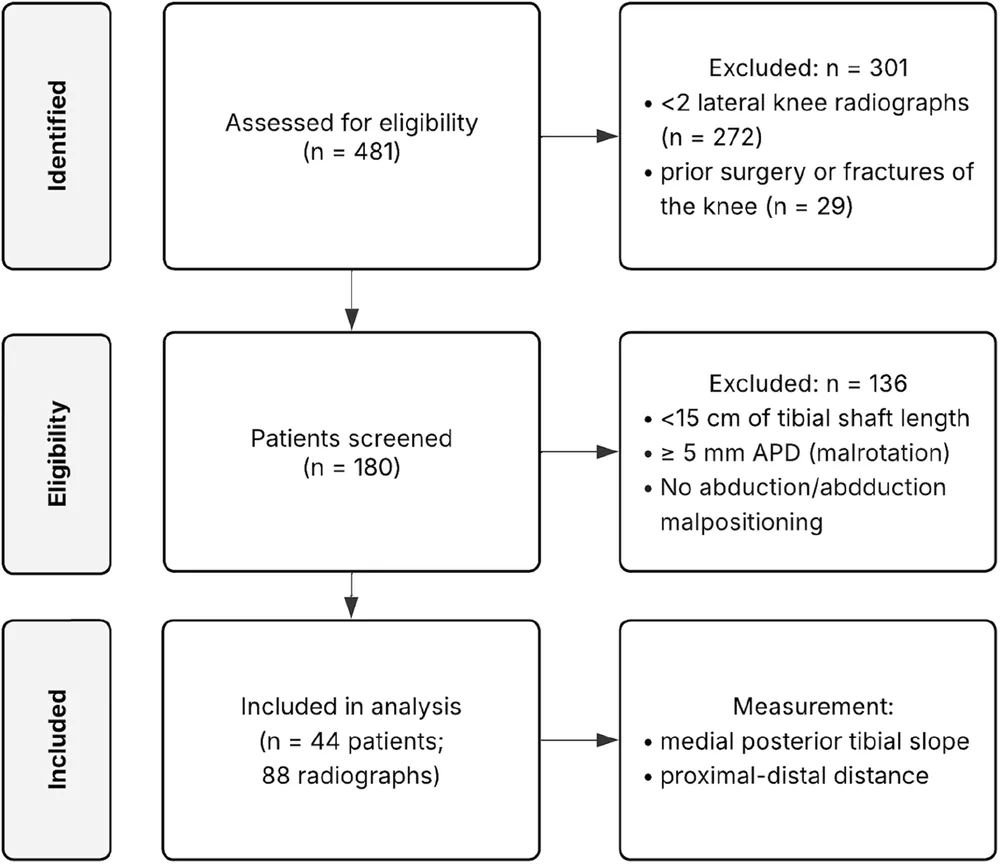
Flow chart of patient selection. APD, anterior–posterior distance.

Demographic data collected included age at the time of surgery, date of radiograph, sex, side, and body mass index (BMI), which were obtained from the patients' electronic medical records.

### Radiographs

Lateral, weight‐bearing radiographs of the affected knee were obtained either during outpatient clinic visits or at the time of surgical indication. Radiographs were acquired using a standardized protocol: the patient was positioned standing, with the knee flexed to approximately 45°, the detector aligned parallel to the sagittal plane, and the center X‐ray beam directed at the patellofemoral joint line.

All images were calibrated using a standard reference marker of 25.4 mm (1 inch). Radiographs were obtained using a digital radiography system (XGEO GC85A; Samsung).

### Measurement technique

Measurement of the mPTS was performed on both the malaligned and optimally aligned knee radiographs, according to the method described by Dejour and Bonnin [7]. To determine the proximal tibial diaphyseal axis, a line was drawn between two points identified at equal distances from the anterior and posterior cortices: one just below the tibial tubercle and another located 10 cm distally. At the level of the tibiofemoral joint, a reference line was drawn perpendicular to this axis. The posterior inclination of the tibial plateau was then measured by drawing a line between the most superior points of the anterior and posterior rims of the medial tibial plateau. The angle formed between this line and the perpendicular reference line was defined as the mPTS (Figure 3). mPTS measurements were recorded in degrees and rounded to the nearest whole degree to reflect clinical practice.

**Figure 3:**
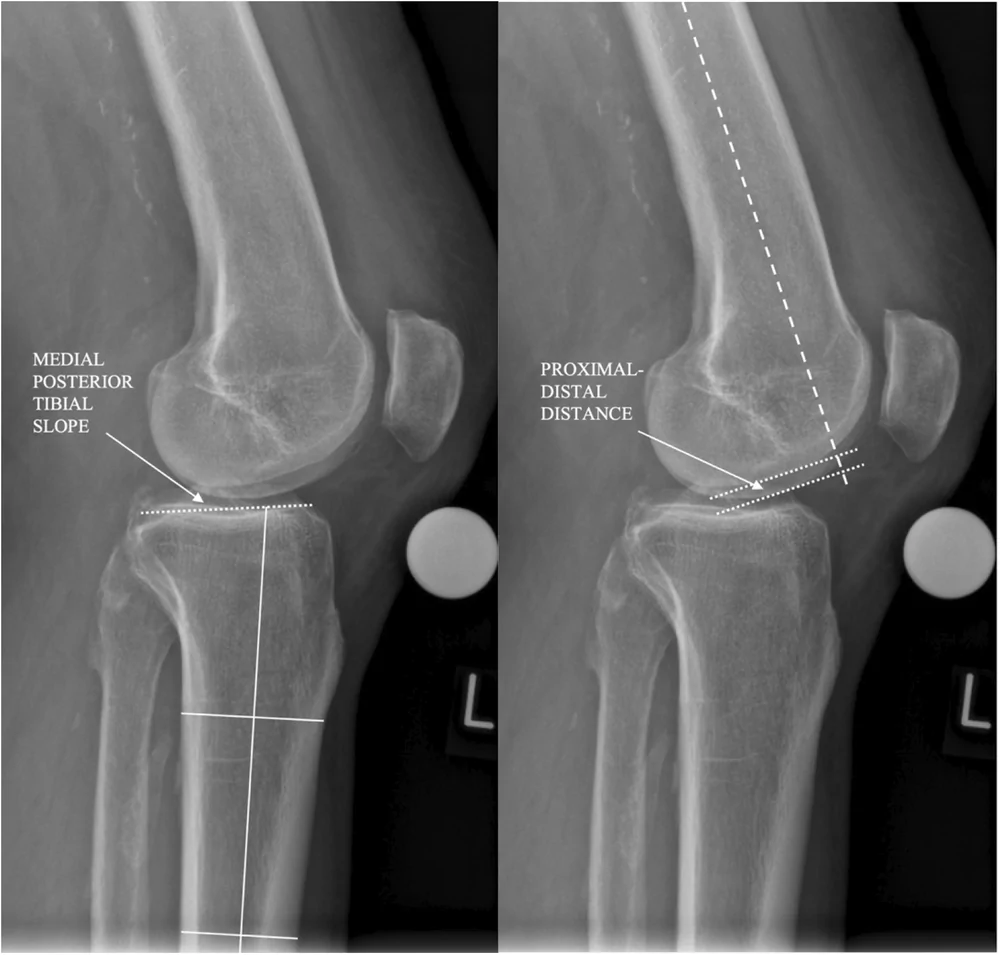
Measurement of mPTS and PDD. Lateral radiograph of a left knee showcasing measurements of mPTS (left) and PDD (right). mPTS, medial posterior tibial slope; PDD, proximal‐distal distance.

Abduction/adduction malalignment was quantified using the PDD of the femoral condyles as previously described [2]. PDD serves as a reliable surrogate, as it correlates with abduction/adduction positioning and has been shown to significantly influence PTS measurements in a sawbone model [2]. Based on Bixby et al.'s experimental findings, demonstrating that a PDD ≥5 mm results in clinically relevant PTS deviations, a cut‐off of 5 mm was used to categorize radiographs into properly aligned and malaligned groups. To measure the PDD, two lines perpendicular to the femoral shaft axis were drawn and positioned as tangents to the most distal points of the medial and lateral femoral condyles. The distance between these lines, measured along a line perpendicular to them, was defined as the PDD (Figure 3). PDD was reported in millimeters and rounded to the nearest whole degree to reflect clinical practice.

All measurements were conducted by two independent observers (M.E. and L.B.), each blinded to the other's results and to patient clinical data, using a PACS workstation (Centricity RIS‐I 4.2 Plus; GE Healthcare). PDD measurements and subsequent view classification were completed prior to mPTS measurements to avoid circularity or recall bias. Reading order was randomized across sessions. To evaluate both inter‐ and intra‐rater reliability, each observer repeated all measurements after a minimum interval of 14 days.

### Statistics

All extracted data were compiled and summarized using Microsoft Excel (version 16.78; Microsoft Corporation). Statistical analysis was performed using IBM SPSS Statistics (version 28.0; IBM Corp.).

To calculate the statistical power achieved by the given sample size, a post hoc power analysis was performed using G*Power (Version 3.1.9.6). Power was calculated at 0.9965 using a two‐tailed *t*‐test, based on an effect size (Cohen's *d*) of 0.72 derived from mPTS means and standard deviations, with an alpha error probability of 0.05.

Descriptive statistics were reported as means and standard deviations (SD). In addition, the magnitude of individual paired differences was analyzed, with results expressed as median and interquartile range (IQR). The mean absolute error (MAE) and root mean squared error (RMSE) were also calculated to further characterize the typical magnitude of deviation. The proportion of cases falling within predefined bounds (±1°, ±2°, ±3°) was additionally reported.

The Shapiro–Wilk test was used to assess normality, and Levene's test was applied to evaluate the homogeneity of variances. For normally distributed variables, comparisons between abduction/adduction malaligned and properly aligned radiographs were performed using two‐tailed paired‐sample *t*‐tests. In cases of non‐normal distribution, the Wilcoxon signed‐rank test for related samples was used. Statistical significance was defined as *p* < 0.05 for all comparisons.

Inter‐ and intra‐rater reliability was assessed by calculating the intra‐class correlation coefficient (ICC), which was categorized as follows: slight (0‐0.2), fair (0.21‐0.4), moderate (0.41‐0.6), good (0.61‐0.8), or excellent (>0.8) [17].

A Bland–Altman analysis was performed to assess agreement between mPTS measurements on radiographs with an abduction/adduction malalignment properly aligned lateral knee radiographs, calculating the mean difference and 95% limits of agreement.

Measurement error metrics were derived from the reliability analysis. The standard error of measurement (SEM) was calculated as SEM = √(SD×1 − ICC). The smallest detectable change (SDC) was then determined as SDC = 1.96 × √2 × SEM, representing the minimal change required to exceed measurement error with 95% confidence.

The percentage of cases classified as outliers, defined as a difference of more than 2° in mPTS between the abduction/adduction malaligned and properly aligned radiograph, was reported. A threshold of ≥2° was chosen because previous studies have identified this value as the minimal clinically important difference (MCID) for PTS measurements [3, 5, 15, 23, 27, 28]. Additionally, it was evaluated whether mPTS values shifted from ≥12° to <12°, or vice versa, when comparing abduction/adduction malaligned radiographs to properly aligned lateral radiographs. This analysis revealed whether patients might have been considered for slope‐correcting osteotomies based solely on malaligned imaging, compared to if radiographs had been repeated. The proportion of patients whose classification changed was reported as a percentage. In addition, a sensitivity analysis was performed focusing on patients with mPTS values in the properly aligned group between 10° and 14°, that is, near the 12° surgical decision threshold. Within this corridor, the proportion of cases that changed classification (<12° vs. ≥12°) when measured on malaligned radiographs was calculated.

## RESULTS

A total of 88 lateral knee radiographs, including 44 with abduction/adduction malalignment and 44 without, met the inclusion criteria. Demographic and clinical characteristics are summarized in Table 1. The mean interval between radiographs was 50.32 ± 33.61 days.

**Table 1:** Patients characteristics.

Parameter	Mean ± SD or no. (%)
Age, years	73.3 ± 8.9
BMI	29.8 ± 5.0
Sex	
Male	19 (43%)
Female	25 (57%)
Side	
Right	22 (50%)
Left	22 (50%)

Abbreviations: BMI, body mass index; SD, standard deviation.

Inter‐rater reliability was excellent, with ICCs of 0.91 for mPTS and 0.94 for PDD. Intra‐rater reliability was similarly high, with ICCs of 0.92 for observer L.B. and 0.93 for observer M.E.

The mean mPTS was 7 ± 3.25° (95% CI, 6.22–8.20) in the properly aligned radiographs and 9 ± 2.52° (95% CI, 8.53–10.06) in the abduction/adduction malaligned radiographs, with a statistically significant mean difference of 2 ± 2.91° (95% CI, 1.20–2.97; *p* < 0.001). The median absolute difference was 2° (IQR 2.31°), MAE was 2.90°, and RMSE was 3.55°. Accuracy‐within‐bounds analysis showed that 13.6% of patients were within ±1°, 40.9% within ±2°, and 63.6% within ±3° of agreement between malaligned and properly aligned radiographs.

The mean PDD was 3 ± 0.17 mm (95% CI, 2.23–2.91) in the correctly aligned group and 7 ± 0.23 mm (95% CI, 6.87–7.78) in the abduction/adduction malaligned group, with a statistically significant difference (*p* < 0.001).

Bland–Altman analysis demonstrated 95% limits of agreement for mPTS measurements ranging from −3.62° to 7.80° between malaligned and properly aligned radiographs (Figure 4). Based on the malaligned group SD (2.52°) and the intra‐rater ICC of 0.93, the SEM was 0.67°, resulting in a SDC of 1.85°.

**Figure 4:**
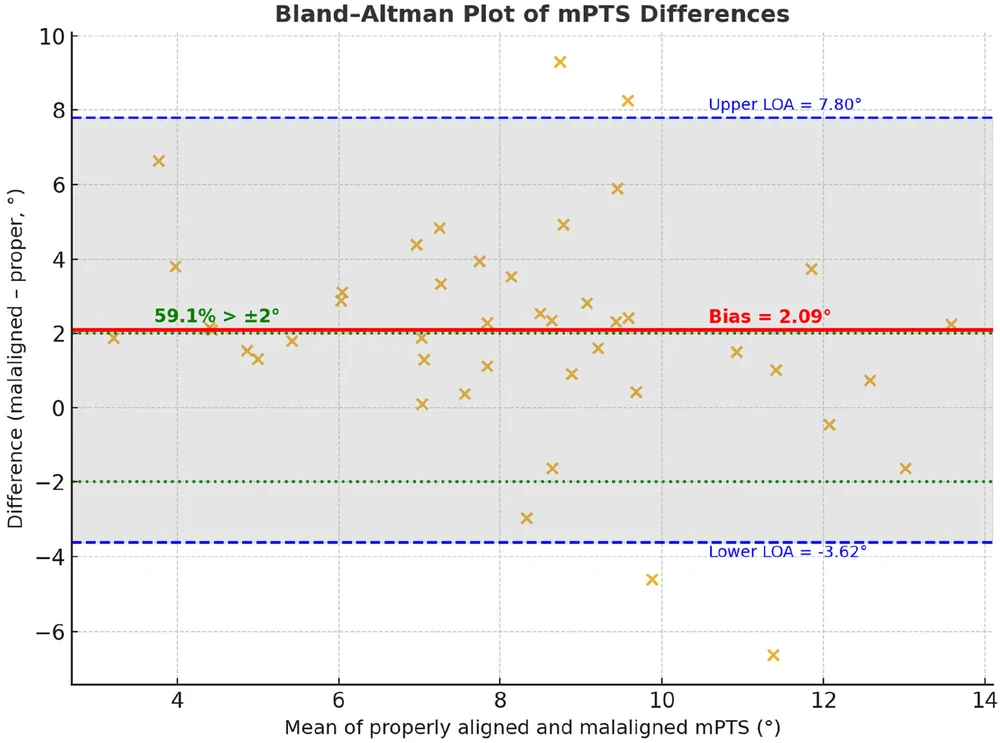
Agreement of mPTS measurement between abduction/adduction malaligned and properly aligned lateral knee radiographs. Bland–Altman plot showing the agreement between mPTS measurements from abduction/adduction malaligned and properly aligned lateral knee radiographs. The mean difference (bias) was +2.09°, with 95% limits of agreement ranging from −3.62° to 7.80°. mPTS, medial posterior tibial slope.

A total of 26 of 44 patients (59.1%) were classified as outliers, defined as having a ≥2° difference in mPTS between imaging conditions. Figure 5 illustrates an example from the same patient, where abduction/adduction malalignment resulted in a 2° higher mPTS.

**Figure 5:**
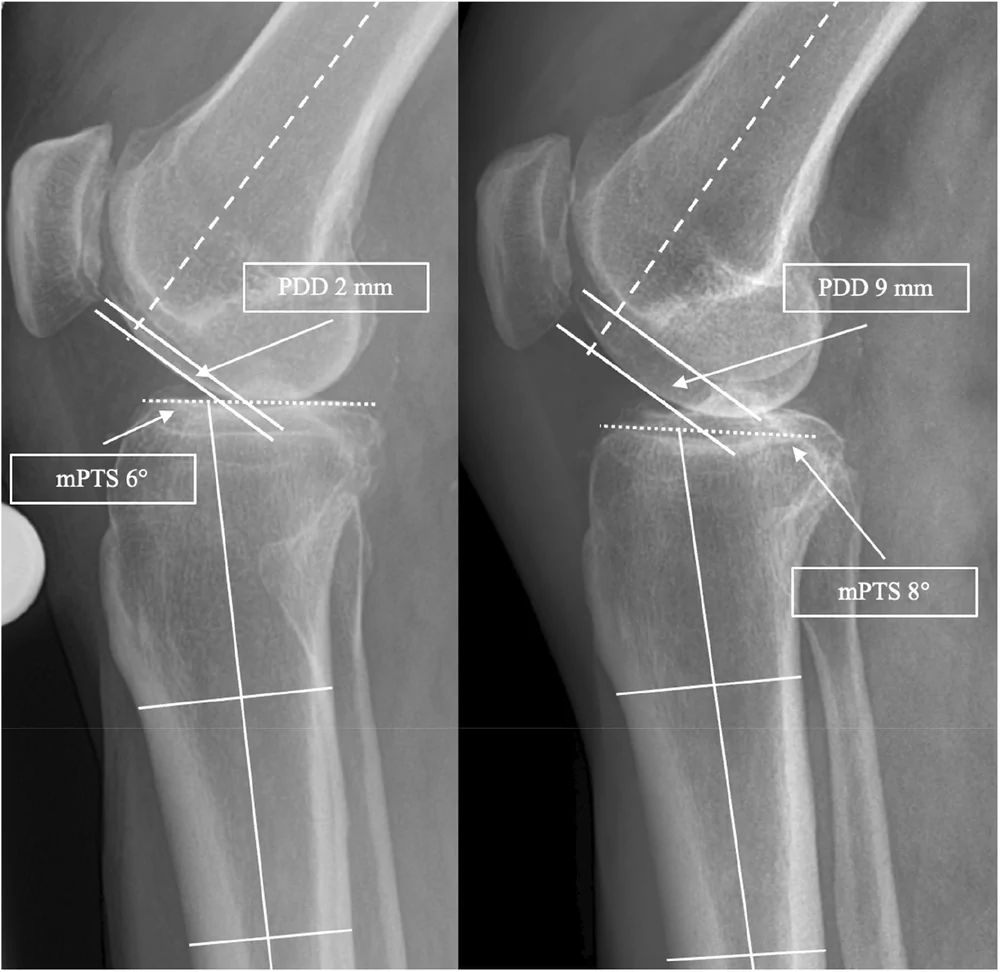
Effect of abduction/adduction malalignment on mPTS measurement in the same patient. Representative lateral knee radiographs from the same patient. Left: properly aligned radiograph (PDD < 5 mm). Right: abduction/adduction malaligned radiograph (PDD ≥ 5 mm). The measured mPTS is 2° higher in the malaligned image, illustrating the systematic overestimation. mPTS, medial posterior tibial slope; PDD, proximal‐distal distance.

Based on the 12° of mPTS threshold, 7 out of 44 patients (15.9%) changed classification: 4 patients shifted from <12° to ≥12°, and 3 shifted from ≥12° to <12° when measured on abduction/adduction malaligned radiographs. In the sensitivity analysis of patients with mPTS between 10° and 14° in the properly aligned group (*n* = 7, 15.9%), 2 patients (28.6%) changed classification relative to the 12° threshold when assessed on malaligned radiographs.

## DISCUSSION

The key finding of this study was that mPTS measurements significantly differed between properly aligned lateral knee radiographs, and lateral knee radiographs with an abduction or adduction malalignment in the same individual. Specifically, malaligned radiographs resulted in a significantly higher mean mPTS, with an average difference of 2° (*p* < 0.001). Bland‐Altman analysis confirmed a wide range of variability, and over half of patients exceeded ≥2° difference. Notably, 15.9% of patients changed classification relative to the 12° mPTS threshold, indicating that reliance on malaligned radiographs could potentially influence the decision to perform slope‐correcting osteotomies. A difference of ≥2° was chosen because previous studies have identified this value as the MCID in PTS measurements. While such deviations may not alter the surgical indication in patients with baseline PTS well below 12°, they become highly relevant when values approach the surgical threshold. These results remained robust after accounting for measurement error (SDC 1.85°) and magnitude‐based indices and were particularly pronounced in patients near the 12° threshold, where nearly one‐third were reclassified. These findings support our hypothesis that abduction/adduction malalignment alters mPTS measurements with the potential to alter clinical management.

Our findings build upon and extend the observations reported by Bixby et al., who were the first to experimentally assess the influence of abduction/adduction malalignment on PTS measurements using a sawbone model [2]. They demonstrated that abduction/adduction malalignment significantly alters both the PDD and the measured PTS, with each 5° increment of malalignment resulting in an average PDD increase of 5.1 mm and a ≥ 3° change in PTS once 10° or more of malalignment was introduced. A moderate negative correlation between PTS and PDD was observed (*r* ^2^ = 0.57), suggesting a linear decline in PTS as the femur shifts from adduction to abduction. Complementing these results, Peez et al. recently confirmed in a biomechanical study that both rotational (internal/external) and coronal (varus/valgus, i.e., abduction/adduction) malpositioning significantly distort PTS measurements. However, the experimental nature of these studies represents a limitation, as it does not fully replicate clinical imaging conditions.

Our study confirms this relationship in a clinical setting using patient radiographs. We observed a significant increase in PDD (from 3 ± 0.17 mm to 7 ± 0.23 mm), which was associated with a corresponding increase in mPTS (from 7 ± 3.25° to 9 ± 2.52°), resulting in a mean difference of 2° (*p* < 0.001). The 5 mm PDD cut‐off we applied was originally proposed by Bixby et al. in a biomechanical study and thus should be interpreted with caution. A stepwise analysis across narrower PDD categories (e.g., <2 mm, 2–4 mm, 6–8 mm) would provide further insight, but our limited sample size precluded meaningful subgroup analysis. Importantly, in our clinical data, deviations above the 5 mm threshold were consistently associated with an average mPTS difference exceeding 2°, which corresponds to the MCID. Moreover, more than half of patients (59.1%) in our cohort exceeded this threshold, underscoring the clinical relevance of abduction/adduction malpositioning.

In our cohort, 15.9% of patients changed surgical classification based on the commonly used 12° mPTS threshold when measured on malaligned radiographs. Notably, this shift occurred even though the mean mPTS values in our cohort were within the range commonly reported for uninjured ACL populations [19, 30, 36]. Given the substantial variability in mPTS across healthy individuals and the lack of consensus on a universal threshold for ‘abnormal’ PTS values [18, 29, 31, 35] our findings highlight how even moderate deviations from ideal imaging conditions can carry meaningful clinical implications, particularly when mPTS values approach thresholds used to guide surgical decision‐making. This underscores the importance of accurate imaging, as even minor malalignment may shift classification, potentially altering treatment plans. It is reasonable to expect that in patient populations with higher baseline mPTS, such as those with ACL graft failure [9, 18, 29, 31, 35], the magnitude and frequency of misclassification due to malalignment would be even more pronounced.

In patients with increased PTS and ACL graft failure, anterior closing‐wedge high tibial osteotomy has gained popularity as a means to decrease translational forces on the revision ACL graft and mitigate the risk of re‐injury [1, 6]. Although high‐level evidence supporting a universal cut‐off is lacking, a PTS greater than 12° is commonly used in clinical practice to guide the indication for slope‐reducing osteotomies [1, 6, 20].

Therefore, accurate measurement of mPTS is essential in both clinical and research settings. Several factors are known to influence the reliability of mPTS measurements, including insufficient tibial length on lateral radiographs, rotational malalignment, and coronal limb alignment [14, 21, 25]. Despite this, abduction/adduction malalignment and its effect on mPTS measurement have received comparatively little attention. Our clinical findings highlight that mPTS measurements are not only sensitive to these positioning errors but may also lead to misclassification near commonly used surgical thresholds.

These findings have important implications for practice: radiographs with abduction/adduction malalignment should not be accepted for surgical planning, especially when mPTS values approach critical thresholds used to guide interventions. Instead, repeat imaging should be strongly considered to ensure accurate mPTS measurement. Furthermore, in academic studies, the degree of radiographic malalignment, specifically PDD and ADD, should always be reported quantitatively rather than described generically to ensure reproducibility and allow appropriate interpretation of PTS. From a pragmatic clinical perspective, we propose a simple workflow when interpreting mPTS. First, radiograph quality should always be confirmed, ensuring PDD < 5 mm, adequate tibial length (≥15 cm), and absence of malrotation (APD < 5 mm). Second, in cases with mPTS values between 10° and 14° (±2° around the 12° threshold), repeat lateral radiographs should be obtained, and confirmation by a second reader or, if necessary, cross‐sectional imaging such as CT or MRI considered. Finally, surgical indications for slope‐reducing osteotomy should not be based solely on a single radiographic parameter but rather on converging evidence, taking into account multiple factors such as imaging results, clinical history, and physical examination.

## LIMITATIONS

This study has several limitations beyond those inherent to the retrospective design. First, only the mPTS was measured, thereby not accounting for potential asymmetry between the medial and lateral PTS. However, the mPTS is more reliably visualized on lateral radiographs and was specifically recommended for measurement in a recent international consensus statement [34]. Second, there are multiple described methods for determining the tibial axis on lateral radiographs, and results may vary depending on the technique applied [5, 8, 18, 32]. Third, the demographic profile of our cohort does not fully reflect the younger population typically considered for ACL or revision ACL surgery, as patients undergoing both arthroplasty and reconstructive procedures were included. Therefore, the generalizability of our findings should be interpreted with caution.

A further limitation is that the radiographs included in this study were selected based on strict exclusion criteria, resulting in a sample with optimized imaging conditions. Only radiographs with sufficient tibial length (≥15 cm) and without notable malrotation (APD of <5 mm [10, 11, 16, 24]) were included, allowing isolated evaluation of abduction/adduction malalignment. However, this limits the ability to assess how combinations of multiple sources of malalignment, such as malrotation and abduction/adduction malalignment, interact and compound measurement inaccuracies. In clinical reality, such overlapping errors are common and may have additive effects on PTS measurements. Therefore, while our findings support a cut‐off of 5 mm PDD as a threshold for acceptable imaging, stricter criteria may be warranted when other image quality issues are present. It should be noted that the 5 mm cut‐off was originally proposed by a biomechanical study and has not yet been validated clinically; we applied it as a literature‐based reference point. In addition, due to the limited sample size resulting from the strict inclusion criteria, we were unable to perform a stepwise analysis across narrower PDD categories. Another limitation is that we did not distinguish between adduction and abduction malalignment, nor did we assess their isolated effects on mPTS measurements, as done by Bixby et al. [2]. Instead, we summarized abduction/adduction deviations using the PDD and categorized radiographs into two groups based on the clinical 5 mm PDD threshold [2]. As a result, we were unable to evaluate the linear relationship between the exact magnitude of malalignment and the degree of mPTS measurement deviation. Further studies should combine clinical and experimental approaches to systematically assess the individual and combined effects of abduction/adduction and rotational malalignment, as well as other factors such as tibial length, on mPTS measurements. This may help establish compound malalignment thresholds that warrant repeat imaging to ensure accurate surgical decision‐making.

## CONCLUSION

Abduction/adduction malalignment significantly alters mPTS measurements on lateral knee radiographs. A clinically relevant difference of ≥2° was observed in over half of patients, and 15.9% changed classification relative to the 12° surgical threshold. Lateral radiographs with a PDD ≥ 5 mm should not be used for surgical planning, and repeat imaging should be considered when mPTS values approach surgical decision thresholds.

## AUTHOR CONTRIBUTIONS

Each named author has substantially contributed to conducting the underlying research and drafting this manuscript.

## CONFLICT OF INTEREST STATEMENT

Alan Getgood: Consultant for Smith and Nephew, Stock ownership in Personalized Surgery and Kyniska Robotics. The remaining authors declare no conflicts of interest.

## ETHICS STATEMENT

Please include the name of the institutional review board (IRB) and the approval number. If not applicable, please state so. The study protocol was approved by the local ethics committee (EA2/016/21), and the study was conducted in accordance with the Declaration of Helsinki. Written informed consent was obtained from all patients included in this study.

## Data Availability

The datasets generated and analyzed during the current study are available from the corresponding author upon reasonable request.
